# Protocolized Brain Oxygen Optimization in Subarachnoid Hemorrhage

**DOI:** 10.1007/s12028-019-00753-0

**Published:** 2019-06-19

**Authors:** Verena Rass, Daria Solari, Bogdan Ianosi, Max Gaasch, Mario Kofler, Alois J. Schiefecker, John-Paul Miroz, Paola Morelli, Claudius Thomé, Ronny Beer, Bettina Pfausler, Mauro Oddo, Raimund Helbok

**Affiliations:** 1 Neurological Intensive Care Unit, Department of Neurology, Medical University of Innsbruck, Anichstrasse 35, 6020 Innsbruck, Austria; 2 Neuroscience Critical Care Research Group, Department of Intensive Care Medicine, Centre Hospitalier Universitaire Vaudois (CHUV), University of Lausanne, Lausanne, Switzerland; 3 Institute of Medical Informatics, UMIT: University for Health Sciences, Medical Informatics and Technology, Eduard Wallnoefer-Zentrum 1, 6060 Hall, Austria; 4 Department of Neurosurgery, Medical University of Innsbruck, Anichstrasse 35, 6020 Innsbruck, Austria

**Keywords:** Aneurysmal subarachnoid hemorrhage, Brain, Critical care, Neurology

## Abstract

**Background:**

Brain tissue hypoxia (P_bt_O_2_ < 20 mmHg) is common after subarachnoid hemorrhage (SAH) and associated with poor outcome. Recent data suggest that brain oxygen optimization is feasible and reduces the time spent with P_bt_O_2_ < 20 mmHg from 45 to 16% in patients with severe traumatic brain injury. Here, we intended to quantify the brain tissue hypoxia burden despite implementation of a protocolized treatment approach in poor-grade SAH patients and to identify the simultaneous occurrence of pathologic values potentially amenable to treatment.

**Methods:**

We present a bi-centric observational cohort study including 100 poor-grade SAH patients admitted to two tertiary care centers who underwent multimodal brain monitoring and were managed with a P_bt_O_2_-targeted protocolized approach. P_bt_O_2_ optimization (≥ 20 mmHg) included a stepwise neuro-intensive care approach, aiming to prevent low cerebral perfusion pressure (CPP), and blood hemoglobin, and to keep normocapnia, normoxemia, and normothermia. Based on routine blood gas analysis, hemoglobin, PaCO_2,_ and PaO_2_ data were matched to 2-h averaged data of continuous CPP, P_bt_O_2_, core temperature, and to hourly cerebral microdialysis (CMD) samples over the first 11 days.

**Results:**

Patients had a Glasgow Coma Scale of 3 (IQR 3–4) and were 58 years old (IQR 48–66). Overall incidence of brain tissue hypoxia was 25%, which was not different between both sites despite differences in the treatment approach. During brain tissue hypoxia, episodes of CPP < 70 mmHg (27%), PaCO_2_ < 35 mmHg (19%), PaO_2_ < 80 mmHg (14%), Hb < 9 g/dL (11%), metabolic crisis (CMD-lactate/pyruvate ratio > 40, and CMD-glucose < 0.7 mmol/L; 7%), and temperature > 38.3 °C (4%) were common.

**Conclusions:**

Our results demonstrate that brain tissue hypoxia remains common despite implementation of a P_bt_O_2_-targeted therapy in poor-grade SAH patients, suggesting room for further optimization.

**Electronic supplementary material:**

The online version of this article (10.1007/s12028-019-00753-0) contains supplementary material, which is available to authorized users.

## Introduction

Besides initial disease severity, secondary brain injury mechanisms largely contribute to the high mortality and morbidity after subarachnoid hemorrhage (SAH) [1]. Multimodal neuromonitoring may help to early identify tissue at risk which may be salvable using appropriate treatment strategies. In severe traumatic brain injury (TBI), a protocolized approach to increase brain tissue oxygen tension (P_bt_O_2_) has recently been shown to be feasible and significantly reduced the time of brain tissue hypoxia to 16% compared to the control group where P_bt_O_2_ < 20 mmHg occurred in 45% [2]. Although underpowered to detect an effect on outcome, the authors proved feasibility and descriptive outcome analysis seemed promising. Brain tissue hypoxia was managed by applying a hierarchical treatment algorithm including optimization of cerebral perfusion pressure (CPP), titration of pharmacologic analgesia, and sedation, adjustment of body temperature, optimization of oxygenation, targeting normocapnia, and red blood cell transfusions (RBC-transfusions) in anemic patients. Similar to severe TBI patients, prolonged brain tissue hypoxia was associated with poor outcome in poor-grade SAH patients [3]. Although P_bt_O_2_-based protocols were implemented in several intensive care units (ICUs), no prospective randomized study supports the use of such protocols. Moreover, it is not clear how high the hypoxic burden remains despite implementation of a protocolized treatment approach after SAH. Therefore, we aimed to analyze continuous neuromonitoring and systemic hemodynamic variables in two university centers using different treatment protocols to decrease episodes of brain tissue hypoxia.

In the current study, we intended to (1) assess the brain tissue hypoxia burden when a strict P_bt_O_2_-guided protocol is applied and (2) to identify factors that are concomitant to brain tissue hypoxia and may be amenable to modification in order to improve brain tissue hypoxia.

## Methods

### Study Design, Setting, and Participants

The study design was guided by the STROBE statement on observational cohort studies. Data of 105 consecutive patients admitted to the neurological ICU at two tertiary care centers (Medical University of Innsbruck = Neuro ICU [NICU] 1 and Medical University of Lausanne = NICU 2) diagnosed with non-traumatic SAH requiring multimodal neuromonitoring between 2010 and 2017 were prospectively collected. Five patients were excluded because of malfunctioning P_bt_O_2_ probes leaving 100 patients for final analysis. Inclusion criteria were (1) spontaneous SAH, (2) age ≥ 18 years, (3) multimodal neuromonitoring of intracranial pressure (ICP), and P_bt_O_2,_ as part of routine clinical care. Invasive multimodal neuromonitoring was initiated in SAH patients requiring prolonged mechanical ventilation and/or clinical or radiological signs suggestive of increased intracranial pressure. The conduct of the study was approved by the ethics committee of the University of Innsbruck and Lausanne (Medical University Innsbruck, AN3898 285/4.8, AM4091-292/4.6; University of Lausanne, CER_VD 2016-01923). Written informed consent was obtained according to local regulations.

### General Clinical Management and Grading

Initial disease severity was clinically quantified using the Glasgow Coma Scale (GCS) and World Federation of Neurological Surgeons (WFNS) Score. Standard treatment conformed to current international guidelines [4, 5]. Ruptured aneurysms were secured by clipping or endovascular coiling. All patients were mechanically ventilated, received appropriate sedation and were continuously monitored for mean arterial pressure (MAP). Prophylactic parenteral (NICU 1) and oral (NICU 2) nimodipine were administered in all patients. Transcranial color-coded duplex sonography (TCD) was routinely obtained in order to follow patients for vasospasm. Vasospasm was defined as elevation of mean velocities > 120 cm/s in the middle or anterior cerebral artery or daily change in mean TCD-velocities > 50 cm/s. Severe vasospasm (> 200 cm/s) was further confirmed by cerebral angiography and treated after interdisciplinary discussion using intra-arterial nimodipine. Delayed cerebral ischemia (DCI) was defined as the occurrence of a new focal neurologic deficit, a decrease of at least 2 points on the Glasgow Coma Scale or a new infarct on the computed tomography (CT) or magnetic resonance imaging scan not attributable to other causes [6]. Functional outcome was evaluated at 3 months post-bleeding by a study nurse blinded to monitor data with the modified Rankin Scale Score (mRS) in NICU 1. In NICU 2, functional outcome was evaluated 6 months after the onset of SAH using the Glasgow Outcome Score (GOS). Outcome was categorized into good (mRS 0–2, GOS 4–5) and poor outcome (mRS 3–6, GOS 1–3).

### Data Collection and Multimodal Neuromonitoring

Baseline characteristics, demographics, hospital complications, and outcomes were prospectively recorded in the institutional SAH databases. Physiologic variables were continuously recorded using the patient data management system (NICU 1: PDMS, CentricityTM Critical Care 8.1 SP2; GE Healthcare Information Technology, Dornstadt German; NICU 2: MetaVision, iMDsoft, Düsseldorf, Germany). Based on clinical and radiological criteria, patients underwent intracranial neuromonitoring including measurement of P_bt_O_2_, ICP, and cerebral metabolites. Neuromonitoring probes were inserted into the hemisphere deemed to be at greatest risk of secondary brain injury either through a frontal burr hole using a triple-lumen bolt or tunneled and placed in the white matter. Probe location was confirmed by CT-scans obtained within 24 h of implantations. In NICU 1, probe location was defined as perilesional in the presence of a focal hypodense or hyperdense lesion within a 1-cm radius of the tip of the P_bt_O_2_ probe, intralesional or otherwise as normal-appearing healthy brain tissue. In NICU 2, all P_bt_O_2_ probes were in healthy appearing brain tissue assessed on head CT-scans. P_bt_O_2_ was measured using Licox^®^ CC1.SB probes (NICU 1: Integra LifeSciences, Ratingen, Germany; NICU 2: Integra Neurosciences, Plainsboro, NJ), ICP by an intraparenchymal probe (NICU 1: Neurovent-P-temp, Raumedic^®^, Helmbrechts, Germany; NICU 2: ICP Codman^®^, Raynham, MA). CPP was calculated by MAP, measured at the level of the foramen of Monro, minus ICP. Cerebral microdialysis (CMD) was performed using a 20 or 100 kD-cutoff-microdialysis catheter (CMA 70 or CMA-71; µDialysis, Stockholm, Sweden). The CMD catheter was perfused with artificial sterile cerebrospinal fluid (Isotonic; Perfusion Fluid CNS; µDialysis AB, Stockholm, Sweden) at a rate of 0.3 µL/min. Hourly samples were immediately analyzed at the bedside for CMD-lactate, CMD-pyruvate, CMD-glucose, and CMD-glutamate using a point-of-care analyzer (ISCUS^flex^; µDialysis AB, Sweden) and frozen at − 80 °C.

### Management of Brain Oxygen

**Fig. 1:**
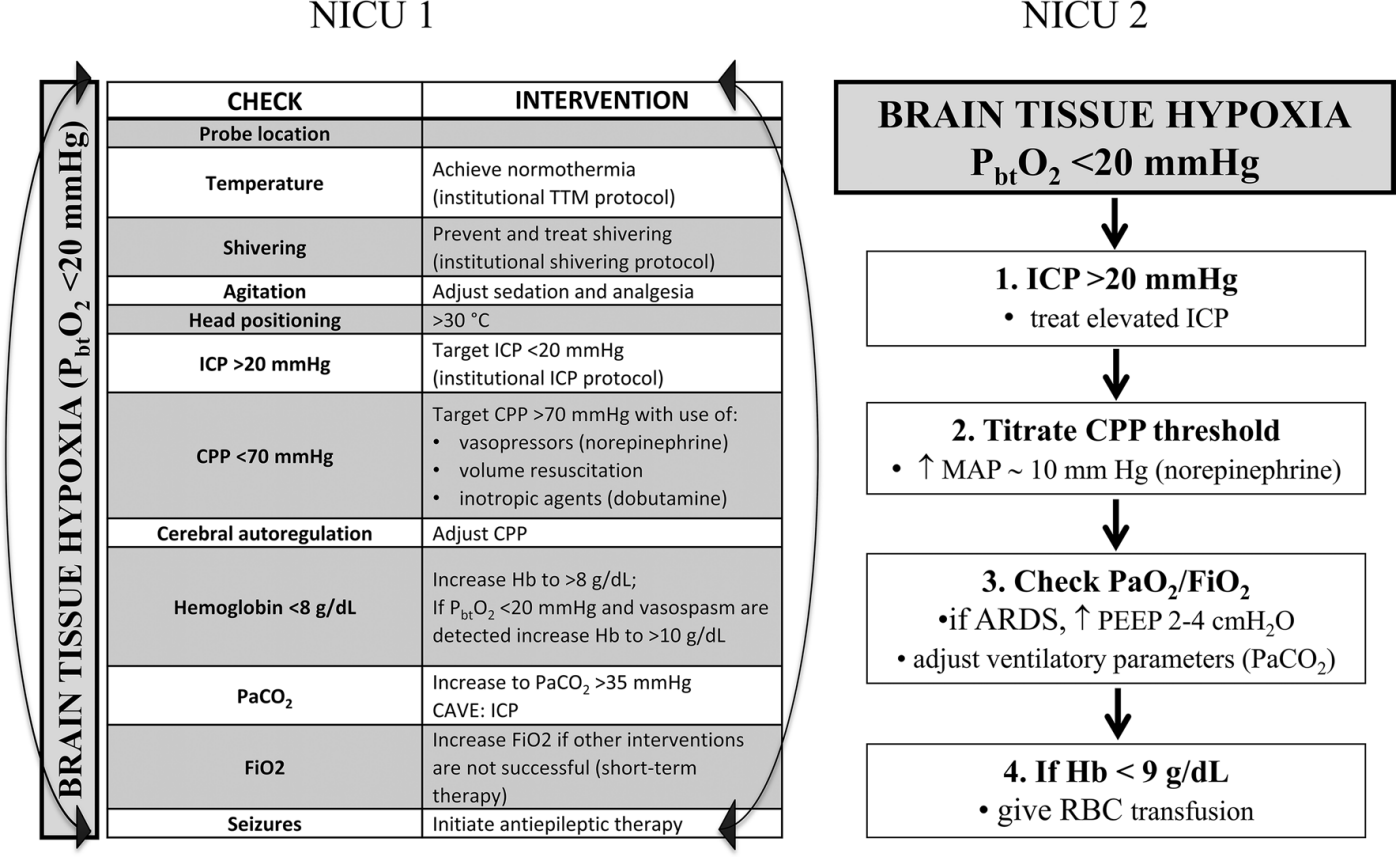
Institutional protocols to treat brain tissue hypoxia (P_bt_O_2_ < 20 mmHg) of NICU 1 and NICU 2

Brain tissue hypoxia (P_bt_O_2_ < 20 mmHg for greater than 10 min) was treated according to institutional protocols (Fig. 1). Treatment options were left to the discretion of the treating neuro-intensivists including optimization of CPP ≥ 70 mmHg with vasopressors if necessary and fluid administration to maintain euvolemia, RBC-transfusions in anemic patients (NICU 1 goal Hb ≥ 8 g/dL, NICU 2 goal Hb ≥ 9 g/dL integrating P_bt_O_2_) and targeting normocapnia (PaCO_2_ ≥ 35 mmHg), normothermia (36.5–37.5 °C) using targeted temperature management, optimization of oxygenation (PaO_2_ ≥ 80 mmHg), and titration of analgesia, and sedation.

### Outcome

The primary outcome parameter of the current study was the incidence of brain tissue hypoxia within the study period. Brain tissue hypoxia was defined as P_bt_O_2_ < 20 mmHg [7].

### Data Management and Statistical Analysis

In order to account for the association of hemoglobin and blood gases and P_bt_O_2_, continuous data of CPP, P_bt_O_2,_ and core temperature as well as hourly measured CMD-samples were averaged over 2 preceding hours and matched to hemoglobin, PaO_2,_ and PaCO_2_ levels derived from routine blood gas analysis. The study period was defined as the first 11 days with the day of admission until midnight denoted as day 0. P_bt_O_2_ values were checked for plausibility and manually cleaned for artifacts, which resulted in exclusion of 5.6% of monitored P_bt_O_2_ data. Hb-levels were categorized into four ranges that approximated the quartiles of their distribution: < 9, 9–10, 10–11, > 11 g/dL. Based on the published literature [2], we predefined variables which potentially qualify for interventions to prevent episodes of brain tissue hypoxia and calculated the incidence during episodes of brain tissue hypoxia over the first 11 monitoring days: CPP < 70 mmHg, anemia (Hb < 9 g/dL), metabolic crisis (CMD-lactate/pyruvate ratio [LPR] > 40 along with neuroglucopenia [CMD-glucose < 0.7 mmol/L]) [8], fever (core temperature > 38.3 °C), PaCO_2_ < 35 mmHg, and PaO_2_ < 80 mmHg.

Continuous variables were assessed for normality and reported as mean ± standard error of mean or median and interquartile range (IQR). Categorical variables were reported as counts and proportions in each group. Groups were compared in univariate analysis using the *t* test, Mann–Whitney *U* test or *Fisher’s exact* test, as appropriate. Brain tissue hypoxia was evaluated as dichotomized as well as continuous variable.

Univariate and/or multivariable generalized estimating equation models were used for all analysis of repeated measurements [9]; with co-variates specified in the results section. Cases with missing values were included. Five patients who were lost to follow-up were excluded from functional outcome analysis.

All analyses and graphical representations were performed with IBM-SPSS V24.0 (SPSS Inc., Chicago, IL, USA) and Prism 5 for Windows V 5.01 (GraphPad Software, Inc., LA Jolla, CA 92037 USA). A *p* value < 0.05 was set as statistically significant threshold.

## Results

**Fig. 2:**
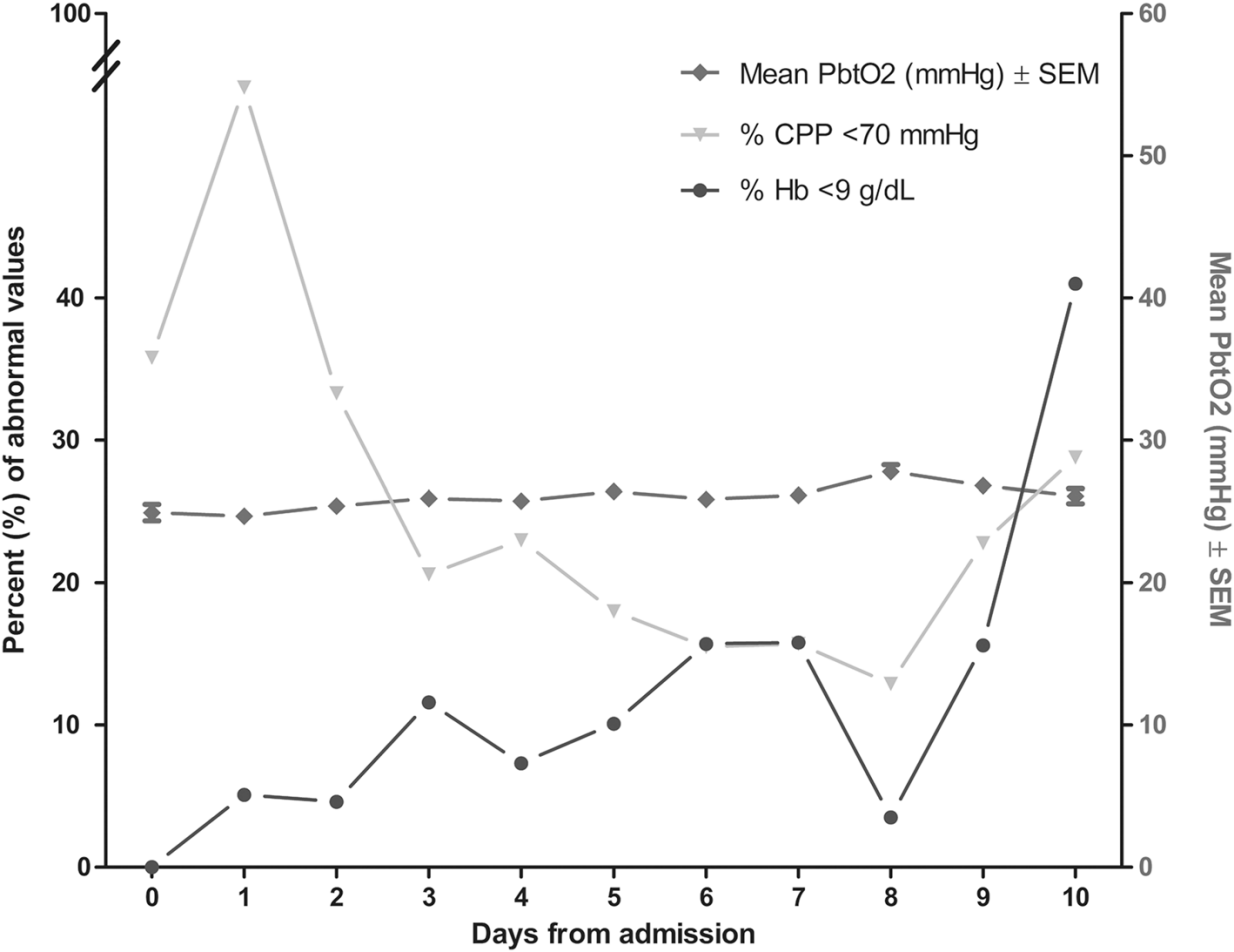
Mean (± SEM) P_bt_O_2_-levels over time are shown. Percentage of anemia (Hb < 9 g/dL) increased, whereas low cerebral perfusion pressure (CPP)-levels (< 70 mmHg) decreased during episodes of brain tissue hypoxia (P_bt_O_2_ < 20 mmHg) over the study period

**Fig. 3:**
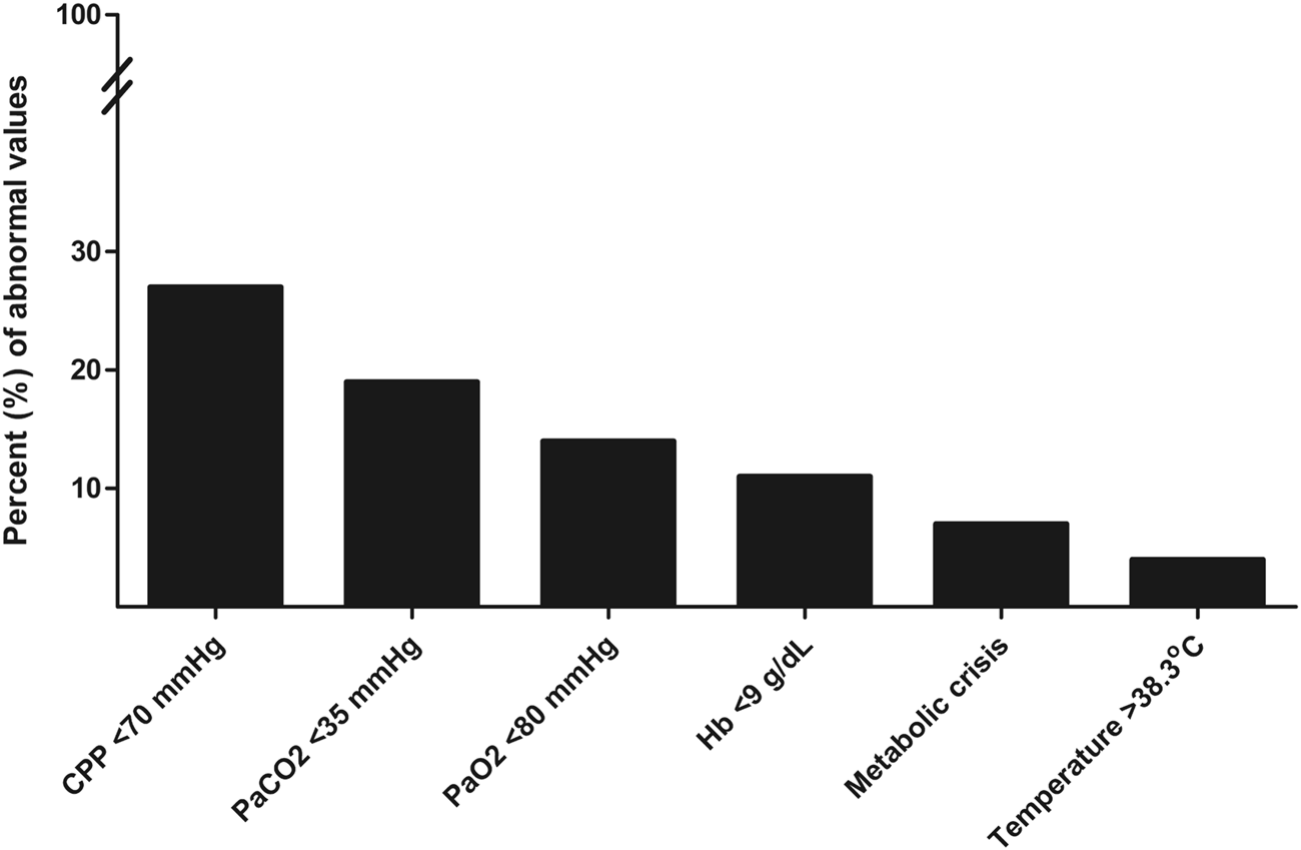
Bars represent the percentage of simultaneous abnormal values shown in the *x*-axis during the time of brain tissue hypoxia (P_bt_O_2_ < 20 mmHg). *CPP* cerebral perfusion pressure, *Hb* hemoglobin

A total of 100 consecutive poor-grade SAH patients were studied. Patients’ baseline characteristics and hospital complications at each site are detailed in Table 1. Altogether, 927 neuromonitoring days with median 11 (IQR 9–11) days per patient were analyzed. This resulted in 5841 analyzed P_bt_O_2_ matched blood gas samples with a median of 7 samples (IQR 4–10) per patient days. No clinically significant complication attributable to P_bt_O_2_ probe insertion occurred. In NICU 1, multimodal monitoring placement associated bleeding was observed in 3/66 (4.5%) patients. However, none of these bleedings was directly associated with the brain tissue oxygen probe. In 5/66 (7.5%) patients, the P_bt_O_2_ probe was contaminated with gram-positive rods without any signs of meningitis, encephalitis or brain abscess formation. Overall mean P_bt_O_2_ was 26 ± 0.1 mmHg and increased over time from 25 ± 0.6 mmHg (day 1) to 28 ± 0.5 mmHg on day 8 (*p* = 0.1, Fig. 2). In 75% of the study time, the targeted goal of P_bt_O_2_ ≥ 20 mmHg was successfully achieved. Episodes of brain tissue hypoxia despite protocolized P_bt_O_2_ treatment occurred in 81% of patients and 25% of analyzed time episodes. The incidence of brain tissue hypoxia was the highest on day 1 (31%) and the lowest on day 9 (20%) (*p* = 0.047). Assessment of predefined concomitant abnormal values revealed low CPP (< 70 mmHg) as most common abnormal factor during episodes of brain tissue hypoxia (27%) followed by PaCO_2_ < 35 mmHg (19%), PaO_2_ < 80 mmHg (14%), Hb < 9 g/dL (11%), metabolic crisis (7%), and temperature > 38.3 °C (4%) (Fig. 3). During episodes of brain tissue hypoxia, CPP < 70 mmHg was most commonly found on day 1 compared to the rest of the monitoring time (*p* = 0.01, Fig. 2). Inversely, incidence of anemia significantly increased over time (*p* < 0.001, Fig. 2). Other potentially treatable factors did not significantly change over time.

**Table 1:** Baseline characteristics, complications and outcome

Clinical characteristics		*N* = 100	NICU 1, *N* = 66	NICU 2, *N* = 34	*p* value
Age in years		58 (48–66)	57 (47–67)	59 (52–64)	0.534
Female sex		72 (72)	46 (70)	26 (77)	0.639
GCS at NICU admission		3 (3–4)	3 (3–7)	3 (3–3)	**0.049**
Admission WFNS Score	1	5 (5)	2 (3)	3 (9)	0.09
	2	11 (11)	6 (9)	5 (16)	
	3	5 (5)	4 (6)	1 (3)	
	4	14 (14)	8 (12)	6 (19)	
	5	63 (64)	46 (70)	17 (53)	
Loss of consciousness at ictus		70 (70)	45 (68)	25 (74)	0.650
*Admission radiological characteristics*					
Modified Fisher Scale	1	2 (2)	2 (3)	0 (0)	**0.008**
	2	4 (4)	4 (6)	0 (0)	
	3	18 (18)	15 (23)	3 (9)	
	4	75 (76)	44 (68)	31 (91)	
Hydrocephalus requiring EVD placement		82 (82)	52 (79)	30 (88)	0.285
Aneurysm size in mm		7 (5–10)	6 (4–9)	7 (5–10)	0.793
*Aneurysm treatment*					
Endovascular coiling		49 (49)	29 (44)	20 (59)	0.199
Neurosurgical clipping		48 (48)	35 (53)	13 (38)	
Non-aneurysmal SAH		3 (3)	2 (3)	1 (3)	1.00
*Complications*					
Pneumonia		69 (69)	47 (71)	22 (65)	0.504
Sepsis syndrome		32 (32)	25 (38)	7 (21)	0.113
Vasospasm		72 (72)	48 (73)	24 (71)	0.818
Delayed cerebral ischemia		38 (40)	19 (29)	19 (56)	**0.001**
Anemia requiring transfusion^a^		29 (29)	18 (27)	11 (32)	0.645
*Outcome characteristics*					
Length of ICU stay in days		25 (16–37)	29 (20–43)	19 (9–25)	**<0.001**
3-month mRS NICU 1	0		4 (6)		
	1		13 (20)		
	2		5 (8)		
	3		8 (12)		
	4		11 (17)		
	5		15 (23)		
	6		10 (15)		
6-month GOS NICU 2	1			14 (45)	
	2			1 (3)	
	3			10 (32)	
	4			6 (19)	
	5				
Poor functional outcome		67 (71)	42 (64)	25 (81)	0.156

Significant differences between NICU1 and NICU2 in univariate analysis (*p* < 0.05) are given in bold

*EVD* extraventricular drainage, *GCS* Glasgow Coma Scale, *GOS* Glasgow Outcome Score, *mRS* modified Rankin Scale, *NICU* neuro ICU, *SAH* subarachnoid hemorrhage, *WFNS* world federation of neurological surgeons

^a^Within study period (11 days)

Absolute mean P_bt_O_2_ levels decreased from 25 ± 0.6 mmHg on day 1 to 23 ± 0.6 mmHg on day 5 in patients with DCI and increased to a maximum of 28 ± 0.8 mmHg on day 8 secondary to induced hypertension with CPP ≥ 70 mmHg (days 6–10: mean 82 ± 0.4 mmHg). Day-wise comparisons of absolute P_bt_O_2_ values showed significantly lower P_bt_O_2_ values in patients with vasospasm on days 2–6 (*p* < 0.001). Similarly, P_bt_O_2_ values were significantly lower in patients with DCI as compared to those without DCI on days 3–6 (*p* < 0.01, Supplementary Fig. 1). Reactive to therapeutic interventions, P_bt_O_2_ increased to a higher level as compared to baseline. Interestingly, we did not find a lower incidence of predefined abnormal values during episodes of normal P_bt_O_2_ (≥ 20 mmHg) as compared to episodes of brain tissue hypoxia.

In NICU 1, 57% of P_bt_O_2_ probes were placed in healthy tissue with overall mean P_bt_O_2_ values in normal range (26 ± 0.19 mmHg), perilesional probe location was recorded in 26% of patients with similar mean P_bt_O_2_ values (27 ± 0.45 mmHg, *p* = 0.473) and intralesional probe location was evident in 7% of patients with significantly lower mean P_bt_O_2_ values (18 ± 0.59 mmHg, *p* < 0.001). In the remaining patients, head CT-scan revealed global cerebral edema (mean 29 ± 0.56 mmHg).

No association was found between P_bt_O_2_-levels and poor functional outcome after 3 and 6 months (adjOR 0.98/mmHg, 95% CI 0.94–1.02, *p* = 0.32) independently of established outcome parameters (WFNS grade, loss of consciousness, age, and anemia, Hb < 9 g/dL) in 95 patients.

### Site-Specific Differences

**Fig. 4:**
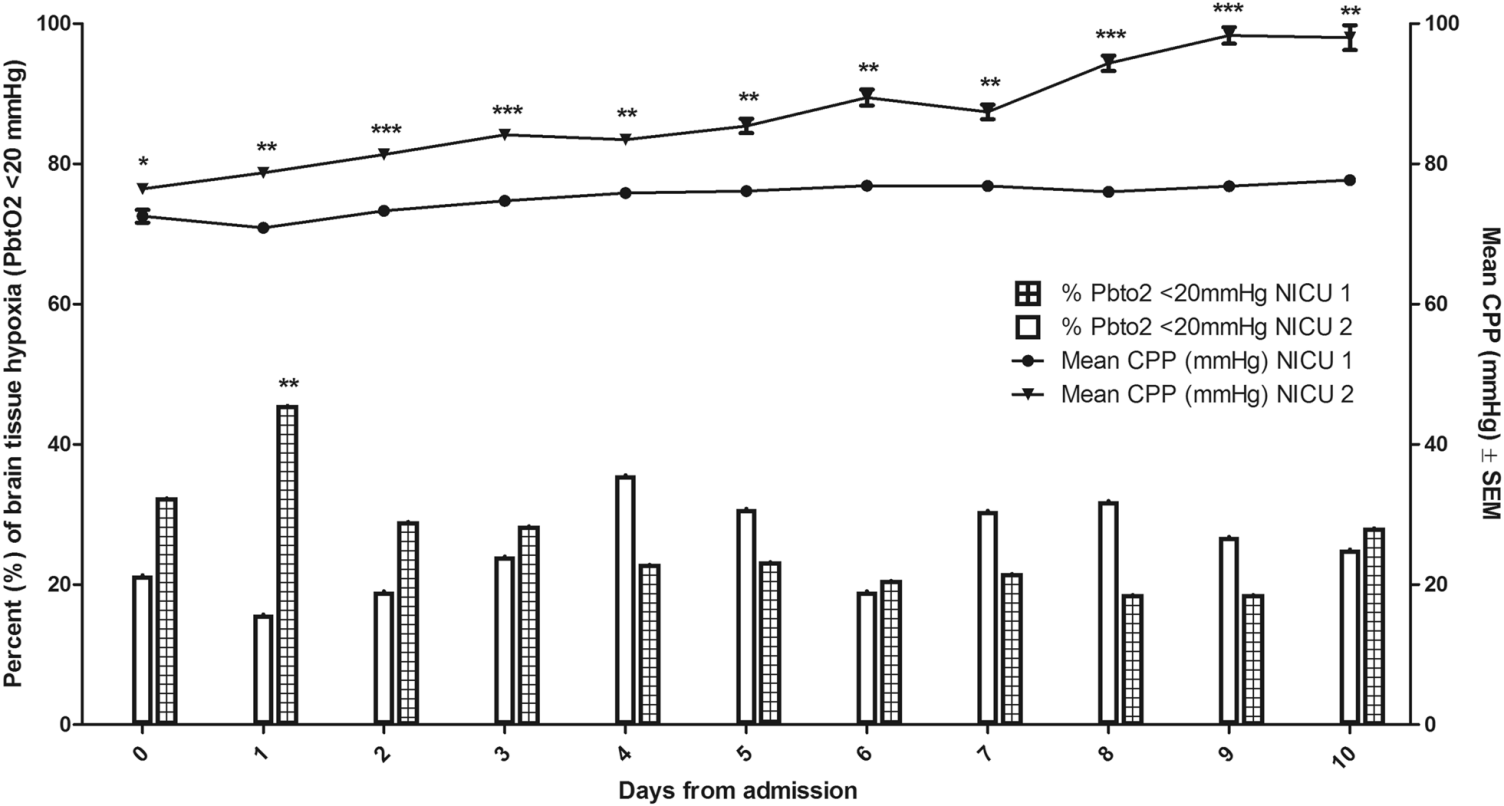
Center-specific mean (± SEM) cerebral perfusion pressure (CPP)-levels and frequencies of brain tissue hypoxia (P_bt_O_2_ < 20 mmHg for at least 10 min) over the study period. *p < 0.05; **p < 0.01; ***p < 0.001

**Fig. 5:**
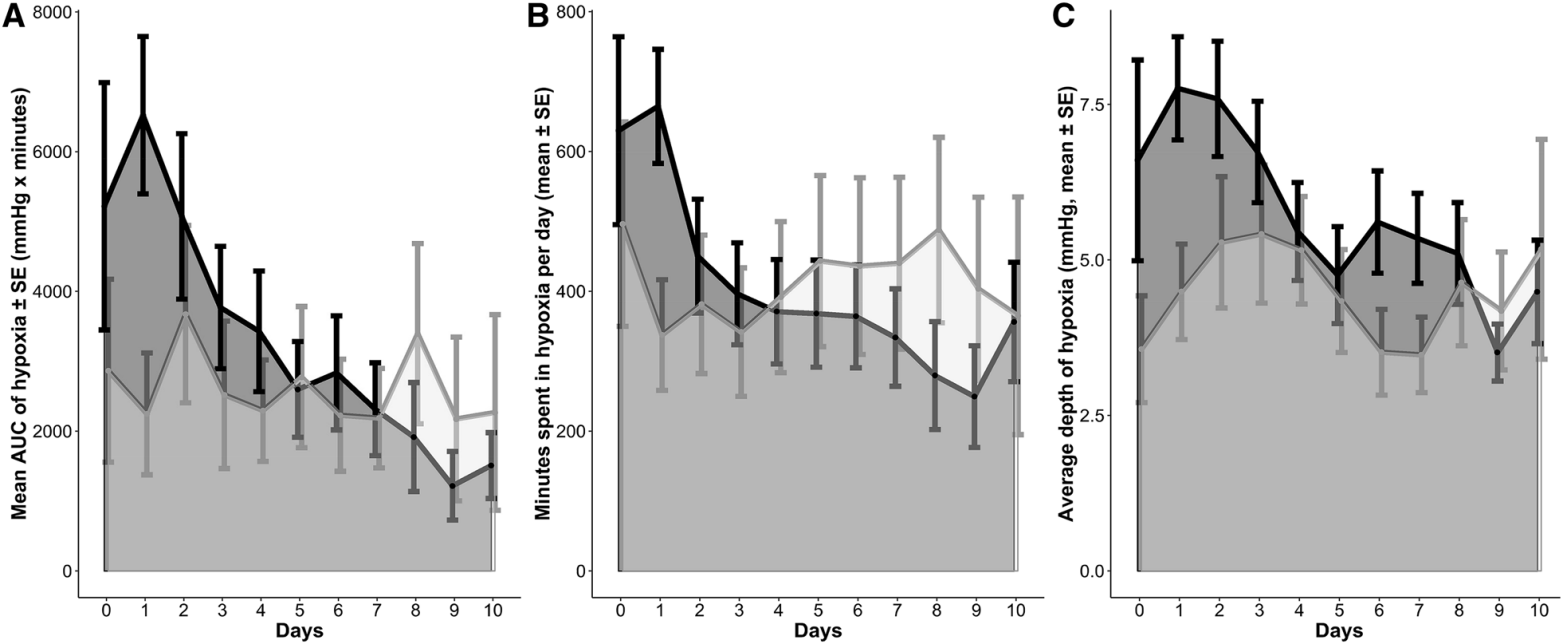
Brain tissue hypoxia (P_bt_O_2_ < 20 mmHg) burden at each site based on P_bt_O_2_ mean values of 5-min intervals. **a** The daily brain tissue hypoxia burden, defined as the mean (± SEM) area under the curve of brain tissue hypoxia (= sum of depth of abnormalities multiplied by the time spent in P_bt_O_2_ < 20 mmHg normalized to monitored time) is reported in mmHg*minutes. NICU 1 is represented by the darker shades of gray, NICU 2 by the lighter shades of gray. **b** Time spent in brain tissue hypoxia expressed in daily mean (± SEM) minutes (normalized to monitored time). NICU 1 is represented by the darker shades of gray, NICU 2 by the lighter shades of gray. **c** Average depth of brain tissue hypoxia (the mean (± SEM) of the P_bt_O_2_ values < 20 mmHg normalized to monitoring time). NICU 1 is represented by the darker shades of gray, NICU 2 by the lighter shades of gray

Patients’ demographics were comparable across the two sites; however, patients admitted to NICU 2 had a lower GCS, a higher mFisher score, and a higher DCI rate (Table 1). Institutional protocols to target P_bt_O_2_-levels above 20 mmHg differed between NICU 1 and NICU 2 (Fig. 1). In NICU 1, interventions to treat brain tissue hypoxia were used without a hierarchical order, whereas a stepwise approach was followed in NICU 2. Moreover, NICU 2 targeted Hb-levels ≥ 9 g/dL and in NICU 1, the goal was ≥ 8 g/dL. Twenty-nine percent of patients were transfused during the first 11 days (NICU 1: 27%, NICU 2: 32%; *p* = 0.65). Mean pre-transfusion hemoglobin levels among transfused patients were higher in NICU 2 (8.6 ± 0.1 g/dL) as compared to NICU 1 (7.8 ± 0.2 g/dL; *p* = 0.001), whereas mean nadir hemoglobin levels in non-transfused patients were 9.1 ± 0.2 g/dL (NICU 1: 8.5 ± 0.2, NICU 2: 10.6 ± 0.3; *p* < 0.001). Similarly, patients in NICU 2 had significantly higher CPP-levels as compared to patients treated in NICU 1 (*p* < 0.001, Fig. 4). In this line, mean-2 h-CPP < 70 mmHg during episodes of brain tissue hypoxia (< 20 mmHg) was lower in NICU 2 (NICU 1: 35%, NICU 2: 8%; *p* < 0.001). Overall brain hypoxic episodes (P_bt_O_2_ < 20 mmHg for greater than 10 min) requiring treatment interventions occurred in 32% (NICU 1: 33%, NICU 2: 29%; *p* = 0.5). Within each patient, the median percentage of P_bt_O_2_ < 20 mmHg (> 10 min) during the study period was 21% (IQR 9–56%) (NICU 1: 24%, IQR 10–73; NICU 2: 19%, IQR 2–44; *p* = 0.09). Day-wise comparison revealed a significantly lower incidence of brain tissue hypoxia in NICU 2 as compared to NICU 1 only on day 1 (*p* = 0.01). Importantly, during DCI-risk time, there was no significant center specific difference in achieving normal P_bt_O_2_-levels despite significantly higher CPP-levels in NICU 2 (Fig. 4). Comparable site-specific differences were obtained for the mean daily AUC (area under curve) of brain tissue hypoxia and time spent in brain tissue hypoxia (Fig. 5).

## Discussion

In this study, we provide clinical data of protocolized P_bt_O_2_-guided therapy to prevent brain tissue hypoxia in poor-grade SAH patients in two independent university centers. In the majority of analyzed time periods (75%), the goal of P_bt_O_2_ ≥ 20 mmHg was successfully achieved. Still, our data suggest that further optimization of systemic variables may be needed. Protocols used in our institutions aimed at optimization of CPP, hemoglobin levels, and sedation depth and maintaining normocapnia, normothermia, and euvolemia which are potential interventions to improve P_bt_O_2_ [2].

Among these factors potentially resulting in low P_bt_O_2_ levels, CPP-values below 70 mmHg were detected most often. In this line, previous studies found an association between CPP < 70 mmHg and a significant higher incidence of brain tissue hypoxia in poor-grade SAH patients [10]. Interventions to increase CPP include hemodynamic augmentation by maintaining euvolemia or induced hypertension using vasopressors [4, 5]. Importantly, we found a lower incidence of episodes with CPP < 70 mmHg during the time when DCI occurred underlining the current recommendation of induced hypertension in patients with DCI. Interestingly, CPP-levels were higher in NICU 2 but did not result in a lower incidence of brain tissue hypoxia during DCI-risk time. In contrast, higher CPP-levels in the initial phase after SAH were related to a higher brain tissue oxygenation in NICU 2. This is of interest and reflects the complex interaction of these variables which are influenced by cerebral autoregulatory capacity (oxygen reactivity index, Orx) [11], neurovascular coupling, CO_2_ reactivity [12], and other factors resulting in increased consumption of local P_bt_O_2_, including spreading depolarizations [13], fever, and seizures. Based on our data, we cannot support the idea of further CPP augmentation without a multimodal neuromonitoring approach in the delayed phase after SAH. Even more interesting, episodes of CPP < 70 mmHg were also found in 25% of time with normal P_bt_O_2_-levels. This may simply reflect that clinicians at both institutions used a P_bt_O_2_-based protocol and accepted lower CPP-values when P_bt_O_2_ levels were within normal range. Finally, it may indicate that de-escalation of CPP augmentation was performed as long as brain tissue hypoxia did not occur.

Delivery of oxygen to the brain tissue is further diminished by hyperventilation which is associated with vasoconstriction of cerebral blood vessels. This is important, since all patients were sedated and mechanically ventilated during multimodal neuromonitoring time. Therefore, PaCO_2_-levels can easily be adjusted by ventilator settings targeting normocapnia or hypercapnia in case of normal ICP. This is consistent with previous findings, suggesting that hyperventilation is frequent after severe brain injury [14] and associated with increased risk for brain tissue hypoxia [15]. In contrast, controlled hypercapnia improved cerebral blood flow and brain oxygenation in a prospective trial [16].

We found anemia (< 9 g/dL) during episodes of brain tissue hypoxia in 11% of observation time. Anemia is a common complication following SAH and has been independently associated with poor outcome [17]. Moreover, an association between anemia and brain tissue hypoxia has been described [18, 19]. RBC-transfusions resulted in increased delivery of oxygen in around 75% of interventions [20, 21]. Some trials suggest improved outcome in patients targeting higher hemoglobin levels [17, 22]. However, the use of RBC-transfusion to correct anemia is controversial due to its potential risks and association with increased morbidity [23]. It is important to mention that site-specific differences with higher Hb-thresholds in NICU 2 were not associated with higher P_bt_O_2_-levels.

We also identified energy metabolic crisis during episodes of brain tissue hypoxia. Elevated CMD-lactate-to-pyruvate-ratio (LPR) together with low CMD-glucose levels in the presence of ischemia suggest increased anaerobic metabolism [24]. This may occur during failure of sufficient energy delivery or increased demand. On the other hand, a high LPR may also result from mitochondrial dysfunction of non-ischemic etiology [25]. Importantly, brain metabolic distress was linked to higher hospital mortality in severely brain-injured patients [26].

Similarly, an increase in body and brain temperature increases brain energy demand. Fever is a common complication after SAH [27, 28] and linked to poor functional outcome [29, 30]. Fever has further been associated with secondary brain injury including vasospasm [31] and DCI [32]. Interestingly, fever was linked to higher LPR-levels indicative of higher cerebral metabolism and presumably increased oxygen consumption in TBI patients [33]. Targeted temperature management with aggressive fever control to achieve normothermia was implemented in both centers resulting in a low overall incidence of fever (6%).

Abnormal modifiable factors during episodes of brain tissue hypoxia may be a target for further improvement in clinical practice. However, treatment adaptations have to be thoroughly considered following an individualized concept in each patient. The natural history of disease (e.g., progressive anemia, DCI) as well as the complex reasons for brain tissue hypoxia is of special importance when using a protocolized approach. This bi-centric study with comparable effects among the two sites despite different treatment protocols suggests generalizability to other institutions following a similar protocol.

### Limitations

Optimization of brain tissue oxygenation is targeted to avoid secondary brain injury including DCI. Furthermore, low P_bt_O_2_-levels may be associated with poor outcome [3]. In our analysis, brain tissue hypoxia was not independently predictive of poor functional outcome. One explanation may be that both centers followed a P_bt_O_2_-guided therapy and a control group were therefore lacking. Second, the dataset was based on blood gas analysis and matched to mean 2-h P_bt_O_2_-levels with limited numbers of analyzed P_bt_O_2_-samples. However, evaluation of P_bt_O_2_ during the whole neuromonitoring time in NICU 1 did not reveal a higher incidence of brain tissue hypoxia and lower P_bt_O_2_-levels were not independently associated with poor outcome (data not shown). The study is further limited by varying daily measurements of Hb-levels within patients (IQR 4–10). An overrepresentation of patients with multiple blood gas analyses per day is possible. However, disease severity did not differ in these patients and P_bt_O_2_-levels were similar (data not shown). The definition of anemia as hemoglobin levels < 9 g/dL irrespective of sex and protocol used is another limitation. However, severely brain-injured patients might have some benefit of higher transfusion thresholds, which is currently investigated in the TRAIN study (ClinicalTrials.gov Identifier: NCT02968654). Moreover, the approach of comparing two centers implicates a certain degree of heterogeneity of how measurements are obtained. Finally, we present a retrospective analysis of prospectively recorded data. Therefore, we cannot provide detailed information about the effectiveness of interventions used to increase P_bt_O_2_.

## Conclusions

This bi-center observational cohort analysis demonstrates that despite the implementation of a P_bt_O_2_-guided therapy, brain tissue hypoxia still occurred during 25% of neuromonitoring time. We found that low CPP, hypocapnia, low PaO_2_, anemia, metabolic crisis, and fever were frequent during brain tissue hypoxia making them to potential targets for interventions to prevent brain tissue hypoxia. Despite applying a protocolized P_bt_O_2_ treatment approach, we could not replicate the previously described association between brain tissue hypoxia and poor outcome after SAH.

## Electronic supplementary material

Below is the link to the electronic supplementary material.

Supplementary Figure 1: Mean (± SEM) P_bt_O_2_ values and frequencies of brain tissue hypoxia (P_bt_O_2_ < 20 mmHg) in patients with and without DCI (delayed cerebral ischemia) over the study period. **p < 0.01, ***p < 0.001 (TIFF 644 kb)

## Abbreviations

SAH: Subarachnoid hemorrhage
TBI: Traumatic brain injury
P_bt_O_2_: Brain tissue oxygen tension
CPP: Cerebral perfusion pressure
RBC-transfusion: Red blood cell transfusion
ICP: Intracranial pressure
MAP: Mean arterial pressure
CMD: Cerebral microdialysis
