# Supplementary figures and images for: Protocolized Brain Oxygen Optimization in Subarachnoid Hemorrhage
Source: Neurocrit Care. 2019 Jun 19;31(2):263–72. doi: 10.1007/s12028-019-00753-0 (PMC6757026; doi:10.1007/s12028-019-00753-0)

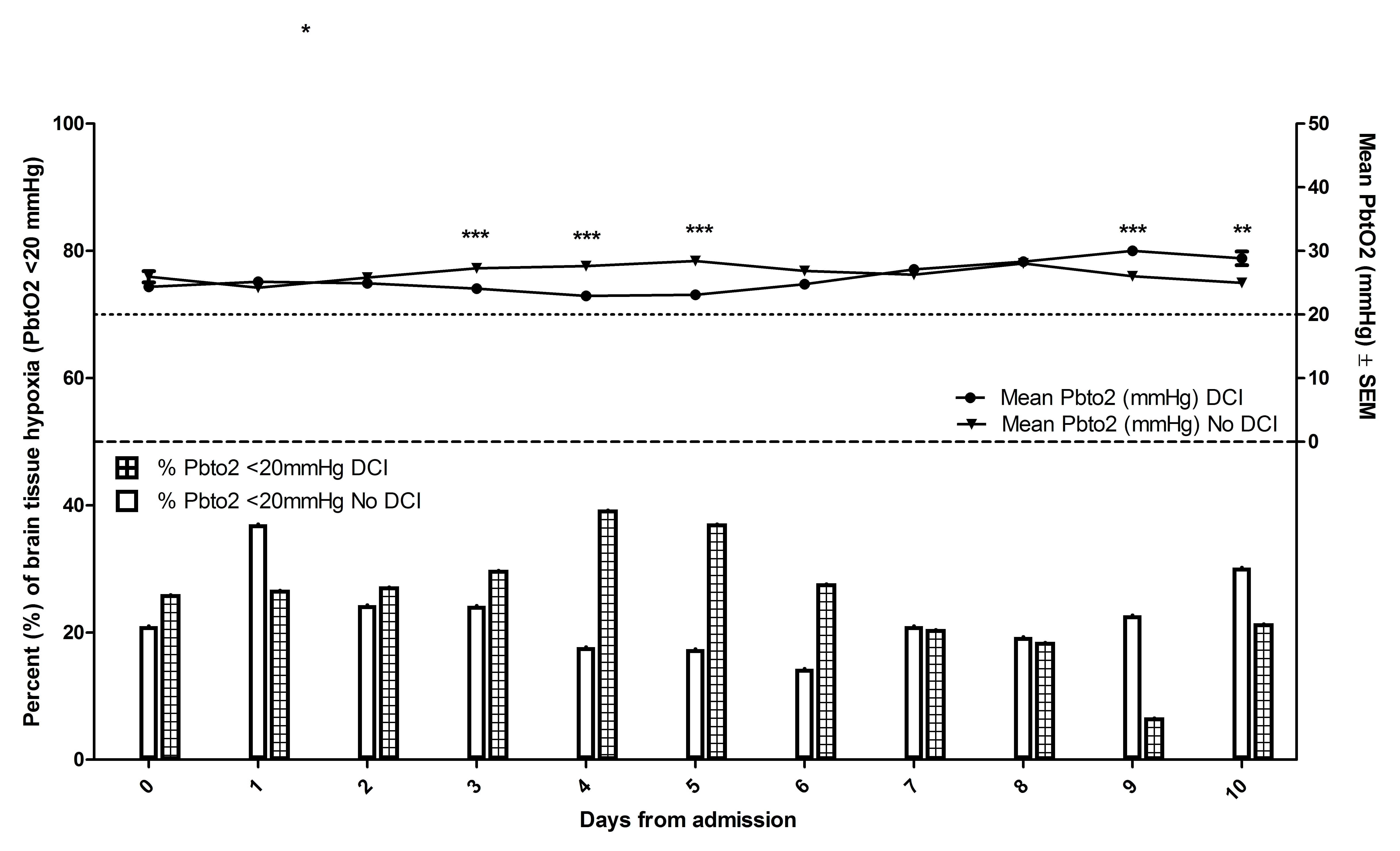

Supplement: Supplementary file 1 — Supplementary Figure 1: Mean (± SEM) PbtO2 values and frequencies of brain tissue hypoxia (PbtO2 < 20 mmHg) in patients with and without DCI (delayed cerebral ischemia) over the study period. **p < 0.01, ***p < 0.001 (TIFF 644 kb) [file 12028_2019_753_MOESM1_ESM.tif]
